# Analysis of Late Antique and Medieval Glass from Koper (Capodistria, SI): Insights into Glass Consumption and Production at the Turn of the First Millennium CE

**DOI:** 10.3390/ma18092135

**Published:** 2025-05-06

**Authors:** Žiga Šmit, Tina Milavec

**Affiliations:** 1 Faculty of Mathematics and Physics, University of Ljubljana, Jadranska 19, SI-1000 Ljubljana, Slovenia; 2 Jožef Stefan Institute, Jamova 39, SI-1000 Ljubljana, Slovenia; 3 Department of Archaeology, Faculty of Arts, University of Ljubljana, Aškerčeva 2, SI-1000 Ljubljana, Slovenia; tina.milavec@ff.uni-lj.si

**Keywords:** glass analysis, natron glass, plant-ash glass, northen Adriatic, PIXE, PIGE

## Abstract

A series (*n* = 22) of glasses from the site Kapucinski vrt (garden of the Capuchin monastery, 5th–17th c. CE) in Koper (Capodistria), a port town in the northern Adriatic, was measured using a combined PIXE and PIGE method. Koper has been continuously populated since the late Roman period, with a rich medieval history, thus offering an opportunity to study Early Medieval glass. Stemmed goblet fragments, in the original publication dated between the 6th–9th centuries CE, and several other vessel types (beakers and flasks or bottles and lamps) were selected for analysis. The measurements were expected to show the trends in glass production and consumption from Late Antiquity until the Middle Ages, notably the transition between natron to plant ash glass and the supply of fresh glass. Among the set of 22 glass vessel fragments, both natron and plant ash glass were identified. For finer classification, we relied on a newly developed method of Euclidean distances with respect to major concentrations. Natron glass of the types Foy 2.1 (9 examples), Magby (2 examples), and Levantine I (Apollonia; 2 examples) was found. Two glasses remain undetermined but testify to an Egyptian origin. Most natron glasses show signs of recycling. Among the three unrecycled glasses (about 20% of the whole set), there are two examples of Levantine glass and a Magby glass lamp; this may indicate a modest supply of fresh glass during the period. Plant ash glass may be attributed to the Early or High Middle Ages, exploiting the purified alkalis of the Levantine coasts (known as *alume catino* in later Venetian glassmaking), and the admixture of impurities in the siliceous sands suggests the circulation and consumption of glass that was produced and traded in the eastern Mediterranean since the 10th century CE.

## 1. Introduction

In glass studies, the 8th–10th centuries CE represent the transition period between the use of natron and plant ash glass compositions. In a broader historical context, this reflects the availability of material supplies and the intensity of trade routes; especially important is the ratio between the old, recycled material and freshly supplied raw glass. In Egypt and Mesopotamia, the production of plant ash glass never completely disappeared. In the Roman world outside this region, it is documented already in the 1st c. CE, though limited to intensively colored blue or green glass [1,2]. A few examples of plant ash glass during Late Antiquity are mentioned in the eastern part of the Roman Empire, such as Crete [3] or Moesia [4,5]. In Italy, the first examples of plant ash or mixed natron-plant ash glass are dated to the 8th c. CE, in Lombardy and around Venice [6,7,8]. An earlier occurrence, such as in the 7th c. CE from Comacchio [9], is opposed in [10]: high MgO values were hypothetically explained as contamination in the crucible. Most of the secondary glass production still used recycled natron glass, at least until the 12th–13th c. CE [7,9,11].

At the same time as the compositions, vessel forms were also changing, especially the stemmed goblet. There is a gap in their development between the ubiquitous Isings 111 form of the 6th–8th c. CE and the appearance of tall-stemmed variants in the 13th–14th c. CE. An opportunity to study the mechanisms of survival of the stemmed goblet during these dark ages is given in the examples from central Italy and the Caput Adriae region. Here, a small group of rare and unique goblet types was documented, usually linked to high-status sites where the demand for drinking vessels and consumer power continued after the 8th c. CE [12,13].

**Figure 1:**
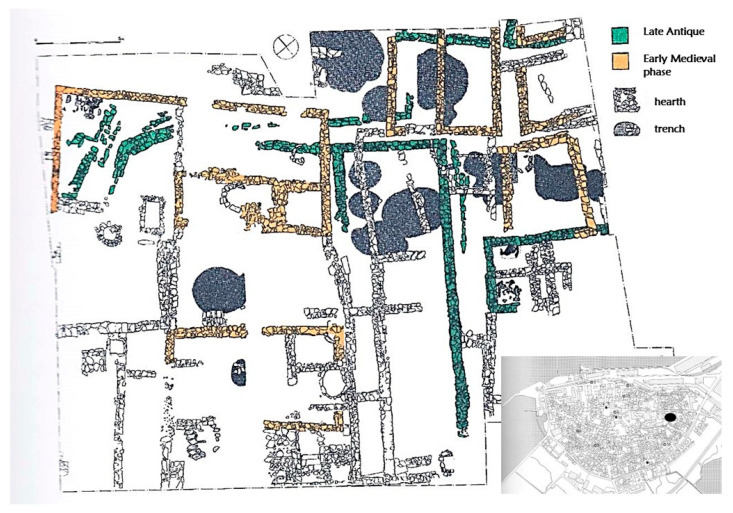
Drawing of the excavation area (after [14]).

To deepen our understanding of the trends in glass production from Late Antiquity to the Middle Ages, we selected 22 samples of vessels from the excavations of the garden of the Capuchin monastery in the port town of Koper/Capodistria in the Slovenian part of Istria. Situated on the Adriatic coast, the archaeology of Koper, previously an island, displays Byzantine and Carolingian/Ottonian influence and, later, a Venetian influence. The garden of the Capuchin monastery revealed a complex stratigraphy of stone buildings from the 5th to the 17th c. CE, when the monastery was built. Until now, only the first two phases, the Late Antique and the Early Medieval phases, have been published, dated from the 5th–10th c. CE (Figure 1). Among the small finds, a 10th c. CE Byzantine belt buckle was discovered in the same building as a coin of Charlemagne, as well as a fitting of a Carolingian spur set [14]. Two illegible dirhems have also been discovered, dated to the beginning of the 9th c. CE, as well as a coin of Constantine VII and Zoe from the beginning of the 10th c. CE [15]. Historical sources report a bishop in Koper (Caprae) in 599 CE. In 908 CE, the Italian king Berengar I promised protection to Adlegida, the abbess of a female cenobium in Koper, named civitas Justinopolitana [16]. A detailed study of the social and political situation of the time revealed that the abbess might have originated from the highest Italian noble families. Caught in the conflicts between the Istrian margrave and the Venetians in the 10th c. CE, the town signed multiple agreements with Venice. Between the 12th and the 13th c. CE, the city prospered as an independent commune, and in 1279, Koper eventually came under Venetian dominance. Historical analysis of the recently available archival sources for 13th–14th c. CE shows the town was comparable to the most important Late Medieval cities on the Adriatic coast, Zadar and Dubrovnik, and that it represented one of Venice’s most important supplying areas. The beginnings of the town’s elevated status can be traced back to the 10th c. CE [17,18].

Excavations in the garden of the Capuchin monastery (45°32′53″, 13°44′03″) in the 1980s uncovered settlement remains dated from the 5th c. CE onwards [14]. They are represented by several houses built in local stone bound with clay and additional buildings built using the post-hole technique (Figure 1). Stone-paved hearths, water channels, and graves of small children were also discovered among the walls. During the excavations, several Isings 111 goblets, lamps, and window glass were found, but also some exceptional Early Medieval goblet types (Cunja types 2 and 4; Figure 4: 1, 3 in ref. [19]). As the site archive is currently under re-evaluation, only a limited amount of glass was offered for analysis by the Regional Museum in Koper. Mainly, goblet feet and stems and parts thereof were chosen for analysis because, among the heavily fragmented material, they could most reliably be assigned to the vessel forms. One sample (22) can be identified as Cunja 2 type. The vessels were given an approximate age estimation (Late Antique: 6th–7th c. CE; Early Medieval: 8th–11th c. CE) according to their stratigraphic position, the phasing of the site published by Cunja [14], and excavation documentation, kept in the Regional Museum in Koper. At the time, the ceramic and especially glass typo-chronologies of the period between the 10th and 13th c. CE were sketchy, and representative objects were not recognized [20]; thus, the end of the Early Medieval phase in the 10th c. CE may have been set too early. A more confident dating of the ceramics from the 13th c. CE onwards allowed the authors to define the Late Medieval phase [21]. The site stratigraphy remains to be published in detail.

**Figure 2:**
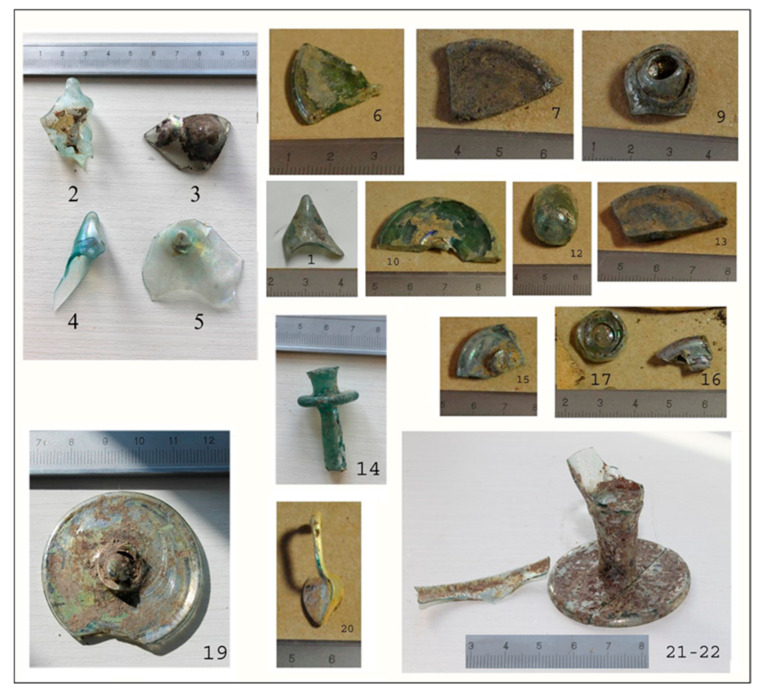
Photos of the analyzed glass. The numbers are the same as in Table 1 and Table 2.

Goblet feet with a small diameter (ca. 3 cm) belong to the Late Antique phase, and the large ones (ca. 4 cm in diameter) to the Early Medieval. This increase in foot size over time is visible in the published goblet types from Koper [14,19] and elsewhere [22,23]. Among the glass materials not published in 1996, a part of a long and thin aqua-colored stem with a disc was discovered and selected for analysis (without context, Figure 2). Comparable tall-stemmed goblets are relatively rare, and in Italy, they are usually dated from 9th–11th c. CE [24,25,26]. Apart from goblets, we also sampled a lamp handle, concave beaker bases, and bottles. We also sampled five pushed-in bases and a bottle or flask rim with a bulge found during the excavation of one of the Early Medieval houses. Still, at the time of sampling, it was estimated to be post-Early Medieval on account of their typological similarity with European Medieval goblet and bottle forms (Figure 2, Table 1).

Our main research question was whether the putative Early and High Medieval goblet types and other vessels were made using natron or plant ash and how our results compare to the other analyses of Early Medieval glass in northern Italy conducted so far.

## 2. The Analytical Method

### 2.1. Experimental

The glasses were analyzed using a proton-induced X-ray (PIXE) and gamma ray (PIGE) method, applying the in-air beam of the Tandetron accelerator at the Jožef Stefan Institute in Ljubljana (HVE, Amersfoort, The Netherlands). The cleanest part of the samples (where oxide layers peeled off upon washing with alcohol) was used for the measurements. The nominal energy of the beam was 3 MeV, but after passing a 200 nm exit window of Si_3_N_4_ and a 7 mm air gap, the impact energy at the target was about 2.94 MeV. The beam intensity was a few nA, and the proton current was measured by an RBS signal from a gold foil on a chopper, periodically intersecting the beam in a vacuum. The measurement of the proton number was checked according to the intensity of the argon signal induced in the fixed air gap between the exit window and the target. As the fluctuations of the signal ratio were below 3%, the chopper measurements were recognized as reliable. Typical measurements were about 30 min per sample. The induced X-rays were detected by a Si(Li) detector (PGT) of 160 eV resolution at 5.89 keV, positioned about 6 cm from the target. The exact distance was determined by measuring a series of elemental and simple chemical compound targets. The detector was further equipped with a pinhole filter made of 0.05 mm thick aluminum foil with a relative opening of 9%; the pinhole transmission function was carefully measured and modeled. The combination of the air gap as an X-ray absorber and the pinhole filter allowed for the detection of X-rays from silicon until antimony in a single spectrum (including, however, the L-lines of heavier elements). The lighter elements, Na, Mg, and Al, were then detected according to their gamma rays, induced by inelastic scattering of protons on the nuclei. The gamma lines used in the analysis were 440 keV for Na, 585 keV for Mg, and 844 and 1014 keV for Al; they were detected by an intrinsic germanium detector (Ortec) of 40% relative efficiency positioned about 10 cm from the target. The concentrations were determined according to the method of fundamental physical parameters for X-rays and according to the surface approximation based on the NIST 620 glass standard for gamma rays, considering the effects of proton stopping and photon absorption simultaneously for both sets of data. The sum of elemental concentrations in oxide form was set to unity, yet for control purposes, it was also compared to the calculated virtual concentration of argon induced in the air gap between the exit window and the target. Departure from the argon nominal value signaled sample mis-orientation or its roughness, which was then considered as a correction in the calculation. The detection limit for Na was about 50 μg/g, for Mg about 0.2%, and for Al about 0.1%. Here, the most critical was the measurement of Mg, on account of low counting statistics of its 585 keV line; obtaining a satisfactory result, thus, regulated the measuring time. The detection limits for X-ray-based elements were about 10 μg/g for mid-Z elements until Z = 30 but worsened to about 50 μg/g around Z = 50 on account of a smaller ionization cross-section. The accuracy of the method, measured according to the NIST 620 and 621 glass standards, was about 5% for major elements but worsened to 10–15% for minor and trace elements.

### 2.2. Determination of Glass Types According to the Euclidean Distance

For the designation of glass types, we designed a numerical method that calculates the Euclidean distance of an unknown glass sample *n* from the multi-dimensional ellipsoid of a specific glass type. The significance of the methods is intended to go beyond the current case; we expected to develop it into a more general tool for the designation of glass types. In the present stage, the Euclidean distances replaced the method of score numbers, tentatively introduced in [27]; this approach often produced undeterminable results, as several glass samples could achieve the same number of scores. A stricter criterion is the Euclidean distance, defined as

(1) $$d^{2}=\sum_{i=1}^{N}{\frac{1}{N}\left(\frac{x_{i}-m_{i}}{t\sigma_{i}}\right)}^{2}$$

where *x_i_* represents the oxide concentrations in the unknown glass, and *m_i_* and *σ_i_* are the mean concentrations and their standard deviations in the specific glass group or series. For a 95% agreement with the specific group, we take *t* = 2. Identification with a specific type is successful if *d* < 1. The ellipsoid has nine dimensions (*N* = 9), considering the major and minor glass composition with the oxides of Na, Mg, Al, Si, K, Ca, Ti, Mn, and Fe, which are reported as most analytical results obtained by different methods. The elements with concentrations around 0.05% and lower, such as Sr and Zr, or trace elements like Li and B, are presently not considered in the calculation but are rather studied in graphs or considered as complementary criteria. It is also important that the number of variables *N* in (1) is not too large, as a disagreement for one selected concentration can be screened by good agreement of the remaining *N* − 1.

The advantage and convenience of the present method is avoiding large sets of individual experimental data that are indispensable in other analytical methods, such as PCA. Instead, the user relies on the elemental averages *m_i_* and standard deviations *σ_i_* of the recognized glass groups that are published by several authors. However, the uncertainty of the method lies in the distribution of individual elements that may deviate from Gaussian values and in incomplete databases.

In the following calculation, we tried to collect databases of different authors that distinguish in the number of collected data and the precision of calculation; especially, the standard deviations *σ_i_* need to be calculated with the same precision as the averages *m_i_* since they define the length scale. Priority was, however, given to the authors who calculated their means and standard deviations from the most complete datasets.

## 3. Results

### 3.1. Elemental Concentrations and Broad Distribution into Groups

The list of samples (Figure 2) with their description is given in Table 1, together with the available stratigraphic/typological dating. Sample selection relied heavily on availability. We are aware of the limitations, but nevertheless, we trust the results are interesting enough to publish them at this stage.

**Figure 3:**
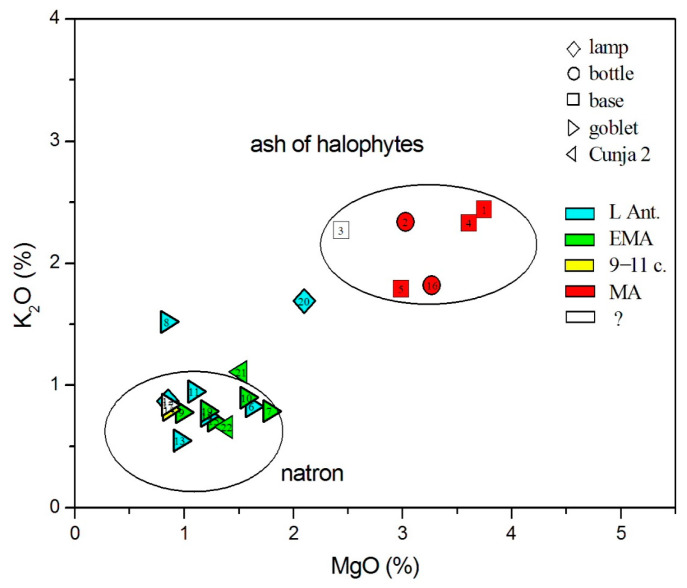
Distribution of measured glasses according to MgO and K_2_O oxides reveals the source of alkalis: natron or halophytic plant ash.

The glasses were first distributed according to the type of flux, which can be resolved from the MgO-K_2_O diagram (Figure 3); natron glass typically has an MgO value below 1.6%, with plant ash above 2.2% [28]. K_2_O values are below 1.5% in natron glass and above 2.2% in plant ash glass [28]. In this and the following graphs, we use different symbols for different glass vessel forms and different colors for the historical periods, as suggested by available typological designations and stratigraphic data. For the vessel shapes, we used diamonds for lamps, circles for bottles, squares for bases, left triangles for goblets, and right triangles for goblets of type Cunja 2. The color scales for the time periods are blue for Late Antiquity, green for the Early Middle Ages, yellow for the 9th–11th centuries, red for the Middle Ages, and white for indeterminable.

Two groups are evident from Figure 3: the natron glass type, which involves both Late Antique and Early Medieval glass forms, as well as the sample dated to the 9th–11th centuries (no. 14), and glass made from the ash of halophytes, which involves presumably post-Early Medieval glass and one chronologically undeterminable example. Only two glasses largely stand apart from the two groups. One is Late Antique goblet no. 8, which shows a somewhat higher concentration of potassium (1.5% K_2_O); however, this value is not exceptionally high and might have been caused by pollution during remelting, where additional potassium might have resulted from contamination from the crucible wall and/or from fuel fume [29,30]. The second sample is Late Antique lamp no. 20, which is found in the intermediate region between the natron and plant-ash glass. This indicates that it was produced either from mixed alkalis or from a mixture of natron and plant ash glass. Its position is also close to the so-called Byzantine Magby glass, as specified in [31].

**Figure 4:**
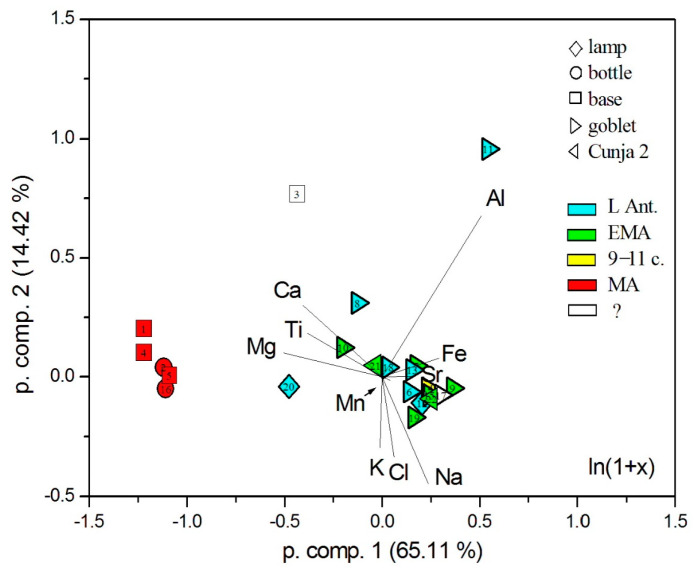
Distribution of the analyzed glasses according to principal component analysis (PCA). The concentrations of 10 elemental oxides plus Cl were logarithmically transformed. The eigenvector of SO_3_ is too small to be shown.

For a broad distribution into groups, we studied the glasses using principal component analysis, considering 11 elemental oxides; to overcome the influence of very large and very small concentrations, we used the logarithmic transform *x*’ = ln(1 + *x*) [32]. Figure 4, again, shows that the presumably earlier glasses (up to the 11th century) form a rather compact group with slightly different samples (nos. 8, 10, and 20). There are two rather different samples: undeterminable no. 3 and beaker no. 11; it is located far in the direction of the aluminum eigenvector, which is due to its high Al_2_O_3_ content. On the other hand, the plant ash glass of the presumably post-Early Medieval group forms an independent compact group.

### 3.2. Natron-Type Glass

The distribution of natron-type glass into groups or series has a complex history; in this work, we will operate with the following terms:

*Roman Mn* and *Roman Sb* [33] will designate pre-4th c. AD Roman glass discolored with Mn and Sb, respectively. These two glasses approximately agree with *Foy Série 3.1 non-tardif* and *Foy Groupe 4* glasses [34] or *RBGY2* and *RBGY1* [35]. The first glass type is of Levantine origin, and the second is of Egyptian origin, respectively. We will not specifically consider naturally colored blue-green-yellow glass [36]. Glass with a higher content of impurities encountered after the 4th c. AD was identified as *Foy Groupe 1* or as *HIMT* (high iron, manganese, and titanium) by Freestone [37], though it was experimentally detected earlier [38]. Several subspecies were identified by several authors, though only *HIMT1* or *strong HIMT* is now recognized as true HIMT. According to its iron content, it is divided into *HIMTa* and *HIMTb* [39]. The other derivatives of HIMT glasses are then rather related to Late Antique glasses of the Foy scheme. Glass *Foy Série 3.2* also involves *HIMT2*, and *Foy Série 2.1* includes *weak HIMT*, *HLIMT* (high lime), and *Ca-rich HIMT* [40]. All HIMT glasses are now considered of Egyptian origin; their Levantine counterparts were designated as *Levantine I* by Freestone and include 4th c. glasses from Jalame and 6th c. glasses from Apollonia [41]. Of the glasses that appear after the 6th century, we considered *Egypt I* (7th to 8th century) and *Egypt II* (8th to 10th century), as well as *Levantine II* (or Bet Elie’zer, 6th to 8th century) [42]. *Egypt II* was split by Schibille into *Egypt 2 (<815 CE)* and *Egypt 2 (>815 CE)* [43]. We further added High Al glasses produced from the evaporitic source of alkalis in Asia Minor [44] and a mixed-alkali Magby glass [45].

The data of the mean elemental concentrations and their standard deviations (*m_i_* and *σ_i_*) are given in Table 3. *For Roman Sb*, they were taken from [46] (Table 1: 269–680 data from refs. [47,48,49,50,51,52,53]. For Roman Mn, we used a compilation [46] (Table 4: 138–239 data from refs. [48,49,52,53,54,55,56,57]. Two subgroups of *Roman Mn* glass are taken from [40]: *Roman Mn—Britain* (7 glasses from [50]) and *Roman Mn—Italy* (12 glasses from [53]). The data for *HIMTa* (14 glasses) and *HIMTb* (5 glasses) are from [58]. The compilation from [46] is used for *Foy Série 3.2* (Table 1: 65–99 data from [34,59,60,61,62], *Foy Série 2.1* (Table 1: 157–180 data from [31,34,58], and *Jalame* (*Levantine I*) (Table 4: 50 data from [63]). Balvanović [64] distinguished two subgroups: *Jalame Mn* (14 glasses from [65]) and *Jalame no Mn* (28 glasses from [65]). Schibille [46] further provides data for *Apollonia* (*Levantine I*) (Table 4: 30 data from [66,67]) and *Bet Eli’ezer* (Table 4, 27–79 data from [41,66,68,69]. Phelps [69] summarizes data for *Egypt I* (24 glasses from [70]) and *Egypt II* (17 glasses from [70]). Data for both Egypt 2 groups are taken from [43] (12 and 24 glasses, respectively, data from [34,41,71], and for Magby, they were taken from [46] (Table 1, 55–65 data from [31,72,73].

The relation between *Egypt II* and *Egypt 2* glasses was inspected from the perspective of Euclidean distances. There is a close relation between *Egypt II* and *Egypt 2 (<815 CE)* (*d* = 0.383), whereas *Egypt 2 (>815 CE)* differs from *Egypt II* (*d* = 4.581) and is closer to *Magby* glass (*d* = 1.336).

**Figure 5:**
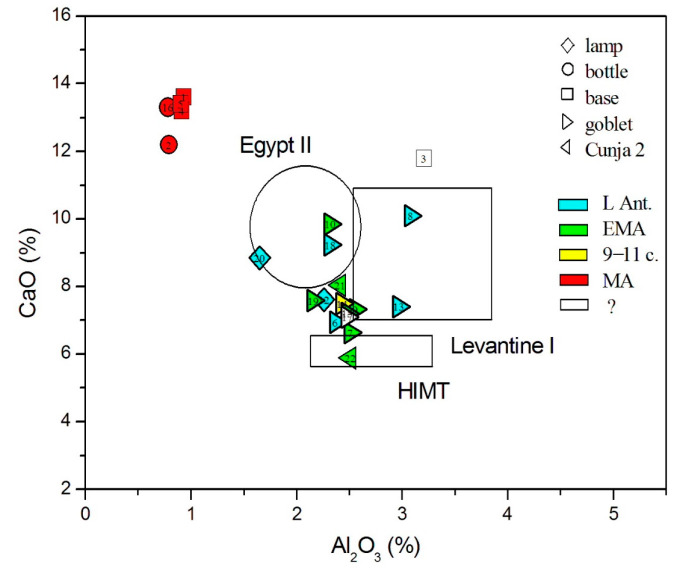
Al_2_O_3_ vs. CaO concentrations approximately distinguish between Levantine and Egyptian sands.

**Figure 6:**
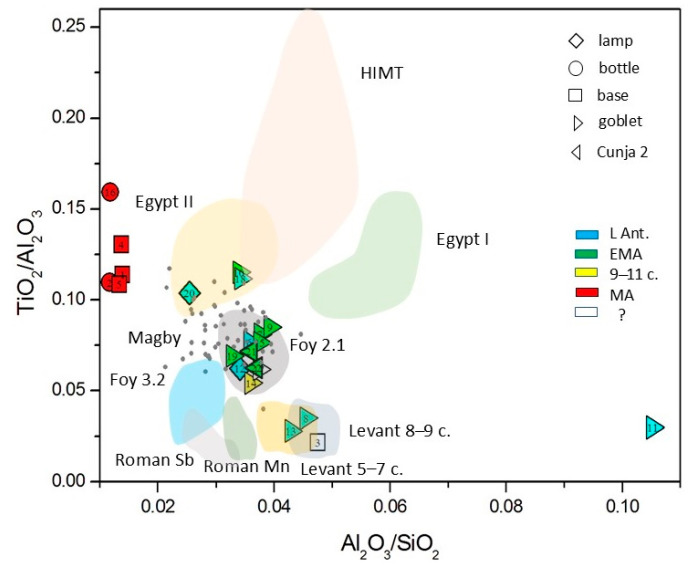
Distribution of measured glasses according to titanium and aluminum oxides. Regions of individual glass types according to the data collected in [33]. Magby data (see references in the text) are added using points.

As natron is chemically a relatively pure agent, the distinction between different glass groups is based on the impurities of the siliceous sand, including aluminum, titanium, iron, and zirconium. The main distinction is between the Egyptian sands, rich in heavy elements brought by the Nile, and the Levantine sands, rich in feldspars, composed of lighter elements such as aluminum. In 2005, Freestone proposed distinguishing glass types according to the Al_2_O_3_-CaO diagrams [42]. In Figure 5, we can distinguish post-Early Medieval plant ash glasses as a separate group, and among the Early Medieval glasses, there are two in the Levantine I area (nos. 8 and 13), one or possibly two are *HIMT* (nos. 22 and 7), and two (nos. 10 and 18 and possibly no. 20 also) are in the *Egypt II* region. All other glasses form a compact group between these groups. As such characterization is now regarded as insufficient, Freestone later [33] presented another diagram, which is based on the Al_2_O_3_/SiO_2_ vs. TiO_2_/Al_2_O_3_ diagram initially proposed by Schibille [57]. In Figure 6, we display our data against the shaded areas of glass types, which Freestone plotted as individual points; additionally, we added the data points of Magby glass [31,45,67]. We can, again, see that the post-Early Medieval plant ash glasses form an individual group, whereas most of the glasses up to the 11th c. form a compact group of *Foy Série 2.1*. There are three glasses in the region of Levantine glass (nos. 3, 8, and 13). Two glasses are in the boundary region between *Egypt II* and *HIMT* glass (nos. 10 and 18), and one (no. 20) seems to be at the other edge of *Egypt II*. There is also an outsider at the high Al side (no. 11).

The conclusions from Figure 3 and Figure 6 are summarized in the second column of Table 4. As a more thorough test, we performed the calculations of Euclidean distances according to Equation (1). The glass type or group with the smallest Euclidean distance is given in the third column of Table 4, and the numerical values of the distances from the group centroids are listed in the fifth column. The respective dating according to the glass type is given in the sixth column of Table 4. The table also marks glasses with apparent signs of recycling.

The results of the calculation of Euclidean distances (Equation (1)) largely agree with Figure 6. Among the natron-type glass, nine glasses were identified as *Foy Série 2.1* and two as *Magby*. The two glasses (nos. 8 and 13) that appear among the Levantine glasses in Figure 4 and Figure 5 are also Levantine, according to the calculation: both are closest to the glass from Apollonia.

The characterization of two glasses (nos. 10 and 18) that, in Figure 6, lie in the region intersecting the areas of *HIMT*, *Egypt II,* and *Magby* glasses is problematic. *Magby* glasses are characterized by the mean values of A_2_O_3_/SiO_2_ = 0.0314 ± 0.0052 and TiO_2_/Al_2_O_3_ = 0.0832 ± 0.0150 (calculated from 53 data points in [31,45,67], while the individual points spread between the upper region of Foy 2.1 and lower region of *Egypt II* (Figure 6). For no. 10, we calculated the following distances with respect to *HIMTa* (*d* = 1.355), *Egypt 2 (d = 1.062),* and *Magby* (*d* = 0.880). For no. 18, we obtain *HIMTa* (*d* = 1.017), *Egypt 2* (*d* = 0.852), and *Magby* (*d* = 0.893). For a distinction between the three types, we further inspect SrO and ZrO_2_ concentrations, which, in both glasses, amount to 300–400 µg/g and about 200 µg/g, respectively. The mean values for *HIMTa* are 519 µg/g and 276 µg/g (from the data of [58], for *Egypt 2 (>815 CE)*, they are 221 µg/g and 244 µg/g, respectively, and for *Magby*, 890 µg/g and 118 µg/g, respectively [46]. Nos. 10 and 18 lie somewhere in between these values and, therefore, cannot be assigned to any definite type. The common property of the three glass types considered is their Egyptian origin. Therefore, we will use the notation *Egypt (?)* for nos. 10 and 18.

### 3.3. Plant Ash Glass

According to the MgO-K_2_O diagram in Figure 3, all post-Early Medieval glasses (nos. 1, 2, 4, 5, and 16) appear to be made of alkalis obtained from the ash of halophytic plants, and among them, the undeterminable base no. 3. Glass no. 20, with its mixed alkali composition, has been determined as Magby glass and is studied among the natron glasses.

Plant ash glass was also subject to the calculation of Euclidean distances (Table 4). For the database, we used the data compilations and measurements from [74] (Tables 11.4 and 11.6), [43] (Table 1), [46] (Table 3), [75] (Supplementary Tables S4 and S5), and [76] (Table 1). Here, the most consistent results were obtained from the data compiled by Phelps, as the plant ash glass nos. 1–5 and 16 were characterized as Tyre (10th–11th c.) or Raqqa (8th–11th c.)—see Table 4; experimental data were taken from [41,77] (8 glasses) and [78] (90 glasses). The distances calculated according to his own data for Ramla (*P1*, *P3*, and *P4*) were greater than unity. According to [43], the classification was Levantine plant ash (data from [78]; 40 glasses)—except for no. 16, which resulted, here, as Mesopotamian due to a slightly smaller distance (*d* = 1.158) in comparison with *d* = 1.166 for the Levantine plant ash. The distances for Egyptian plant ash glasses *E1–E4* [46] were greater than unity. According to the data collected in [75], the plant ash samples also appeared to be made in Tyre (13 glasses 8th–12th c.) according to the data in [77]; however, this classification was a consequence of a very large data spread of values [77], as a large σ make the distances smaller. If we consider as a potential source all distances to be smaller than unity, possible proveniences also include Ctesifon (9 glasses from [78]), Raqqa 1 (91 glasses from [78,79]), Bayreuth (7 glasses from [78]), Raqqa 4 (74 glasses from [78,79]), and Siraf Main A 9th–12th c. (15 glasses from [80]—for no. 2 only). According to the data compiled and calculated in [76], glasses 1–3 were determined as Raqqa 1 (database of 103 glasses from [79]). For glasses 4, 5, and 16, Raqqa 1 remained the second closest, though smaller distances were obtained for Khirbet al Minya (no. 4, *d* = 0.651; database of 6 glasses from [78]) and Sagalassos (no. 5, *d* = 0.612; no. 16, *d* = 0.611; database of 11 glasses from [81]).

Though these locations are quite diverse, most of them are on the Mediterranean coast or its close background, with three exceptions: Sagalassos in Asia Minor (glass could have traveled there by trade), Siraf in Iran (encountered as a modest possibility for glass no. 2), and Ctesifon near Baghdad (attribution to this site may be due to the large standard deviation from the reference concentrations).

## 4. Discussion

### 4.1. Natron Glass

**Figure 7:**
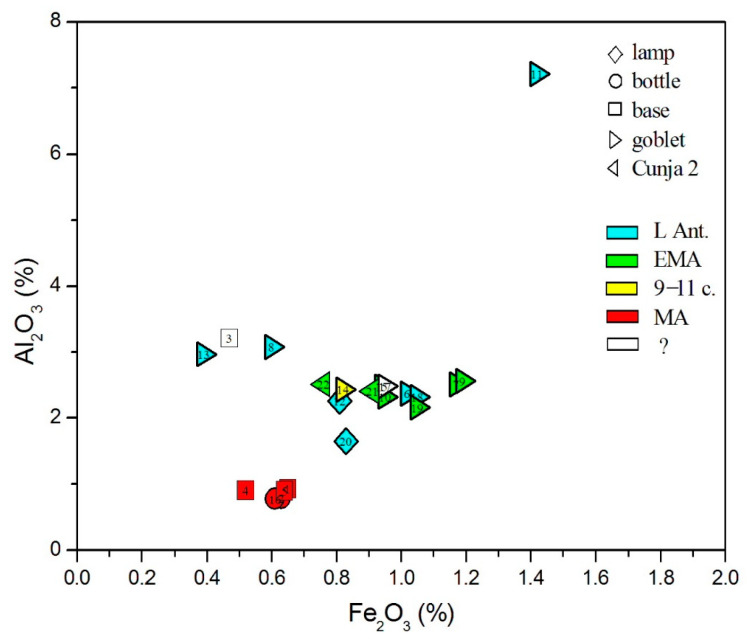
Distribution of glasses according to iron and aluminum oxides.

**Figure 8:**
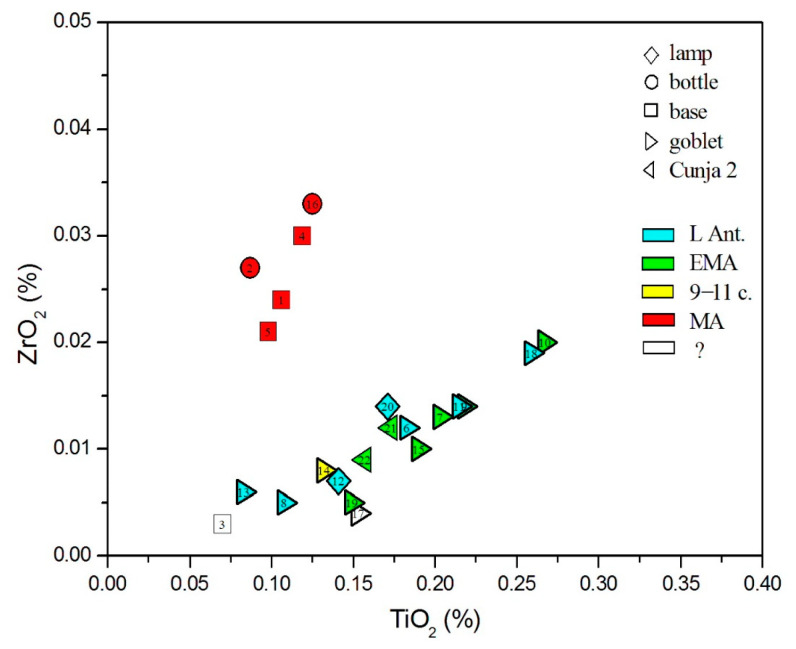
Distribution of glasses according to titanium and zirconium oxides.

Natron-type glass of Late Antiquity was produced in two regions sufficiently close to exploit the dry deposits of Egyptian lakes, Egypt itself, and the Levantine coast. The two regions producing primary raw glass differ according to the impurities in the siliceous sand. Figure 7, showing Al_2_O_3_ vs. Fe_2_O_3_, clearly distinguishes between Levantine and Egyptian sands: Levantine (nos. 8 and 13) is characterized by higher aluminum values and smaller iron content. The situation is similar in the TiO_2_ vs. ZrO_2_ plot (Figure 8). Glasses of Levantine sands (nos. 8 and 13, as well as the plant ash glass no. 3) show both low titanium and zirconium values. Higher values of both elements, showing a linear correlation, are perceived in Egyptian sands, with the highest values in the undetermined Egyptian glasses of nos. 10 and 18. Glass 20, which is made of mixed natron and plant ash alkalis, and glass 21 are then among the Egyptian glass, in accordance with their characterization as Magby glass.

**Figure 9:**
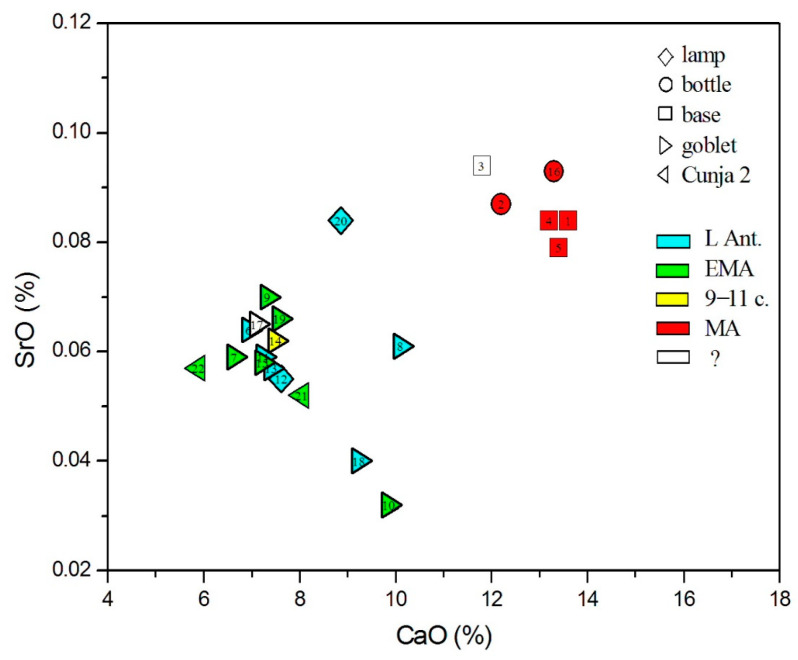
Strontium oxide content with respect to calcium oxide.

Strontium can be used to distinguish mineral sources of calcium from their source in mollusks or plant ash [42]. Figure 9 (showing SrO vs. CaO) reveals that SrO concentrations are typically larger than 300 µg/g, which excludes a mineral source of calcium. The lowest SrO values are observed in the two Egyptian glasses of nos. 10 and 18. Though these values are closest to Sr concentrations in HIMT glass, such classification can be excluded on account of major composition. Juan de Ares [72] noted the structured distribution of *HIMTa* and *HIMTb* glass in the eastern and western Mediterranean, with the absence of temporally later (beginning of the 5th c. CE) *HIMTb* in certain regions, including the northeastern Mediterranean. This may indicate a limited supply of HIMT glasses since the beginning of the 5th c. CE, caused by specific political or economic events. In the region of Koper, this overlaps with changed supply routes, as reflected in amphorae imports. Until mid-5th c. CE, North Africa predominates as the export region, while in the 6th c. CE, most ceramic imports arrive from the Eastern Mediterranean. This change follows a decline in Tunisian pottery workshops, most probably linked to wine and oil production and circulation dynamics [82].

**Figure 10:**
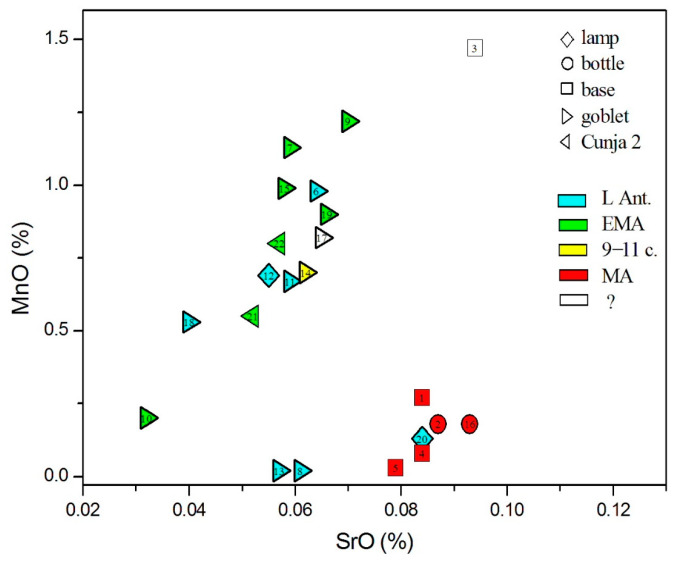
Glasses according to manganese and strontium oxides.

Manganese can enter glass either as an impurity or as a decolorizer added intentionally, for example, in the form of pyrolusite (MnO_2_). In Figure 10 (showing MnO vs. SrO), it is evident that two Levantine glasses (8 and 13) were made of glass that was not discolored with MnO, while glass no. 3 exhibits the highest MnO level of 1.47%. Of the two Egyptian (?) glasses, one (no. 18) exhibits 0.53% MnO, while for no. 10, its content is only 0.2%; for this reason, no. 10 departs from *HIMTa* more than no. 18.

It is further important to consider the percentage of recycled/non-recycled glass. There are several criteria for the distinction of recycled glass: the content of antimony below the level that ensures discoloration [52] and the admixture of heavy elements that enter the glass batch through the colored glass. In our glasses, we did not detect antimony and tin (the detection limit for both elements was about 50 µg/g), which means that the recycling process did not involve a significant amount of glass discolored with antimony and glass opacified with tin. Therefore, we could only rely on the admixture of heavy elements Cu, Zn, and Pb, the values of which in the recycled glass are typically above 100 µg/g, though Zn values may be slightly below this value even in the recycled glass [9]. According to these criteria, all natron glasses in our set are recycled, except for both Levantine glasses (8 and 13). Another example includes lamp 20 (Magby), produced using mixed alkalis; the siliceous component points to this being non-recycled; this finding makes the possibility of mixing natron and plant ash glass less probable.

Compared to vessel typology, both non-recycled Levantine glasses are goblet feet with a diameter of ca. 3 cm, found in the Late Antique (6th–7th c. CE) phase layers at the site. The typology, stratigraphy, and composition of these two samples fit very well. One Magby glass is a lamp handle (no. 20); here, again, the stratigraphy, typology, and glass composition fit. The second Magby (no. 21) is the rim of a further undetermined vessel, quite possibly a goblet or a lamp. It was found together with sample no. 22, a Cunja 2 goblet of recycled Foy 2.1 composition, on a stone floor of the Early Medieval phase. Recycled Foy 2.1 glasses are represented mainly by goblet feet with a diameter of ca. 4 cm or by parts of goblets (nos. 9 and 17) and one lamp or balsamarium. They belong to the Early Medieval phase of the site. Among them are the Cunja 2 type goblet (no. 22) and the thin-stemmed goblet stem (no. 14). As to where they were produced, it is, of course, not possible to give a definitive answer. Still, it seems worth stressing that Cunja type 2 goblets are very similar to a goblet from the workshop at Torcello, now dated to the 9th c. CE or even slightly later [22]; Figure 46. They are both composed of two parts, with a hollow stem and a narrow knob at the top of the stem. The thin-stemmed goblet (no. 14) confirms the reuse of old glass until the 10th or 11th c. CE in the workshop where it was made. To our knowledge, these types of goblets are not found in the same layers as the sturdier Cunja 2 and 4 types or similar vessels. They are also more widely found in the Italian Peninsula, and their development continues into the Middle Ages. It may well be that they were produced in different workshops and, more importantly, in different social and political contexts of the 10th c. CE and later. The second half of the 10th c. marks the beginning of the consolidation of the Ottonian Empire and the rise of the power of Venice. More political stability allowed the now firmly established elites to also pursue their demand for luxury tableware. It also allowed the workshops to meet this demand with a slightly more constant supply than in the two centuries before, yet still relying on the circulation of recycled material. A high percentage of recycled earlier glass was also detected in medieval sites from Italy, such as in Nogara [83], Vetricella [84], or the medieval castle of San Giuliano [85], while fresh Levantine glass still reached Islamic Sicily [86].

Glass no. 11 is a fragment of a concave beaker base, which was found on a layer of fired clay and ash, probably a hearth of the Early Medieval phase. It contains a low level of MgO, which points to a mineral source of alkalis, but it contains a high amount of aluminum. The reason for the high aluminum content is not clear; if we exclude surface pollution, high-Al glasses may be associated with the production in Asia Minor exploiting evaporitic mineral sources of soda [44]. Their characteristic also involves boron and lithium contents, the presence of which we could detect as gamma lines at 429 keV and 478 keV, respectively [87]; however, the two lines in this object could not be discerned from the background in our measured spectra. Using another set of data ([76] and unpublished results), the detection limits for Li and B were estimated to be 10 µg/g and 300 µg/g, respectively. The range of both elements in 11 glasses from Asia Minor is between 16 and 438 µg/g for Li and between 657 and 1810 µg/g, so both elements could have been observed. On the other hand, agreement with the major composition of high-Al glasses is quite good (*d* = 0.636). Another possible origin is glass produced in central Asia and used for beads [88], which is characterized by a high K_2_O concentration and low CaO and SrO concentrations. No. 11 does not match these properties, so the question of its origin remains open.

### 4.2. Plant Ash Glass

Plant ash glasses are produced from cleaner silica sources, as the vegetal ash contains oxides of both alkali and earth-alkaline elements, as well as several impurities. Investigations of the silica matrix usually determine the cleanliness of the silica source [89] or connections with the geological background according to neodymium isotopes [90]. Significant differences are then sought according to the plant ash component.

The Euclidean distances show little differences between the plant ash glasses of our samples and do not allow distinction within quite a broad region involving present-day Lebanon and Syria, with a small probability including Mesopotamia and Asia Minor. As glass was a trading material within the Islamic-Byzantine world, we explore the relation between our glasses and the glass cullet from the shipwreck of Serçe Limani, sunk around 1025 CE. The Euclidean distances according to the mean values of Serçe Limani glass ([76] using 99 data from [63]) calculated for plant ash glasses nos. 1–5 and 16 vary between 0.637 and 0.837. These values show a high similarity between our samples and the traded glass samples.

As Venice was also a renowned trading city in the Mediterranean world, closely interacting with Constantinople and North Africa [91], we compared our glasses (nos.. 1–5, 16) with the Venetian soda glass: greenish-brown and uncolored, from the 11th–14th centuries, and later 15th and 16th c. *Venetian commune* and *vitrum blanchum* glass according to the data compiled by Verità ([92], Tables 6.2.3 and 6.2.4). The smallest distances in the range 0.500–0.658 were obtained for the uncolored glass of the 11th–14th centuries (except no. 3, which was closest to the green-blue glass of the same period; *d* = 0.345). The distances for the *commune* glass were in the range of 0.655–0.834, and the distances for *vitrum blanchum* were in the range of 0.738–0.988. For glass no. 3, both distances were greater than unity (1.047 and 1.528, respectively). Summarily, the mean distances were 0.563 for the glass of the 11th–14th centuries, 0.797 for the later *vitrum commune*, and 1.017 for *vitrum blanchum*, respectively. These values argue strongly towards earlier dating of our plant ash samples and point to the type of glass that was produced in the Levantine area and the matter of extensive trade since the 10th c. CE.

**Figure 11:**
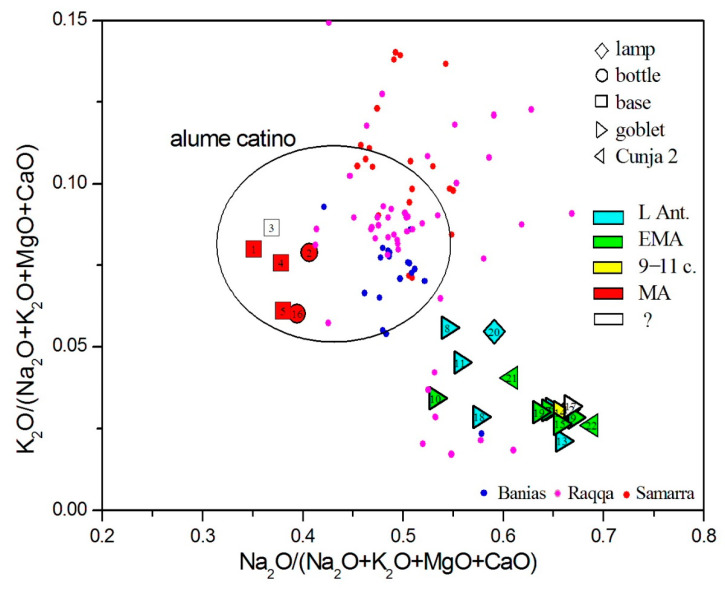
Relative fraction of sodium and potassium oxides reveals the source of plant ash alkalis. Smaller symbols show published data for Banias [41], Raqqa [79], and Samarra [78].

Next, we inspected the properties of the alkali component. The quality of the plant ash can be monitored from the diagram that shows the relative fraction of sodium and potassium oxides in the total sum of alkaline and earth–alkaline oxides (Figure 11). All samples are sorted within an area that, in our previous works, embraced certain fractions of glass from Ljubljana and Celje in Slovenia, Antwerp in Belgium, and Lezha in Albania [93,94]. Within this group, there are also glasses with original Venetian provenance, and according to the conclusion of de Raedt [95], they are made of the finest plant ash, named *allume catino* in the 15th and 16th c. CE, in Venetian glass making, which was harvested along the Levantine coasts. All our post-Early Medieval glasses were also made of alkalis matching *allume catino*; we do not encounter any glass made of lower quality alkalis harvested elsewhere. Measurements of strontium isotopes suggest two production areas for harvesting halophytic plants in the Levant: coastal areas and the interior around the Euphrates River [96]. In Figure 11, we also plotted data for Banias (a representative of the coastal region; data from [41]), Raqqa (a region around Euphrates; data from [79]), and Samarra (a region around Tigris, towards the Zagros mountains; data from [78]). The plot shows differences between the coastal alkalis and those harvested inland (the Raqqa and Banias datasets contain some natron-type glass as well). Our data agrees better with the centroid of the Banias values, confirming that alkalis from the coastal region were more frequently used in the maritime trade.

**Figure 12:**
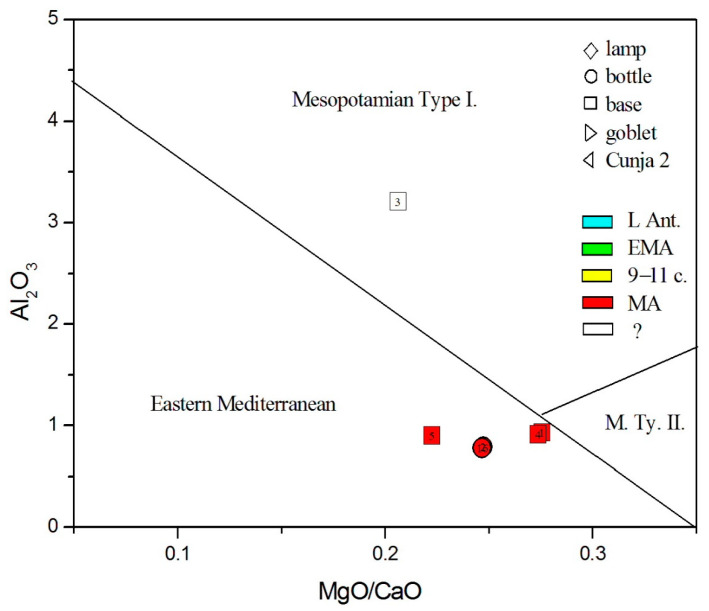
Distinction between Mediterranean and Mesopotamian glass according to Al, Mg, and Ca oxides from [74].

Considering the origin of siliceous sand material, we, again, rely on the admixtures of light and heavy elements, as presented in Figure 5, Figure 6, Figure 7, Figure 8, Figure 9 and Figure 10. In all these figures, all post-Early Medieval glasses appear as a compact group, suggesting their common origin. In Figure 5 (CaO vs. Al_2_O_3_), the post-Early Medieval glasses exhibit the lowest Al_2_O_3_ (below 1%) and highest CaO values (above 12%). High CaO implies high SrO values, between 700 and 930 µg/g (Figure 9). Sample no. 3 departs the group due to its high Al_2_O_3_ concentration, which, in Figure 6, puts it among Levantine glass. The local origin of plant ash glass is also evident in the MgO/CaO vs. Al_2_O_3_ diagram, according to [74]. Figure 12 shows that all post-Early Medieval glasses are made of Eastern Mediterranean ingredients, while the indeterminable no. 3 is rather *Mesopotamian Type I*. The Euclidean distance for no. 3 is inconclusive and is larger than unity, both for Levantine and Mesopotamian glass (*d* = 1.317 for Nishapur). It also bears a resemblance to the Egyptian *E4* glass (*d* = 1.297), though details about this type of glass, dated to 1035–1149 CE, are not yet clear [46]; in Figure 12, *E4* would be characterized as *Mesopotamian Type I.*

All post-Early Medieval samples have an MgO/CaO ratio between 0.2 and 0.3. This ratio was also studied in [96]: MgO/CaO values around and below 0.3 refer to glasses from Banias, Ramla, Beirut, and Damascus, i.e., to the glasses from the Levantine coast. This confirms the hypothesis that the exported glass mainly came from the coastal area [96].

We also consider the possibility that the post-Early Medieval plant ash glasses (nos.. 1, 2, 4, 5, and 16) were imported to Koper from Venice, which mastered the Mediterranean glass market in later centuries. For this, we inspected the minor and trace elements. The content of TiO_2_ varies between 0.087% and 0.125%, and ZrO_2_ between 210 and 330 µg/g. Both elements are then present at concentrations that highly exceed the values for original Venetian production. De Raedt [95] set the zirconium limit at 30 µg/g for a distinction between the imported Venetian glass in Antwerp and domestic production. Similarly, original Venetian glass, according to [97], should not exceed the concentrations of 2% Al_2_O_3_, 0.07% TiO_2_, and 40 µg/g ZrO_2_. Since our titanium values exceed these limits moderately, while the zirconium values considerably, our glasses were not produced from glass using siliceous sands of Venetian glassworks. This would imply they were imported to Koper from a site different from Venice. However, as Venice also imported large amounts of raw glass or its raw materials from the Levant and elsewhere [92,98], it is also possible that the objects were manufactured in the Venetian or other workshops from the imported raw glass or cullet.

High Zr values reopen the question of imports from Egypt, as its prehistoric glass contains significantly high values of zirconium. However, high Zr values are also found in glass from Islamic Ramla, where raw glass chunks were imported from Mesopotamia and Iran during the Abbasid period [74]. This complies with our previous statement that the plant ash from Koper relates to the locations of present-day Palestine, Lebanon, and Syria, as well as possibly Mesopotamia and Asia Minor, in contrast to Egypt.

The presence of six plant ash glass samples in Koper also fits well into the picture sketched by Italian colleagues, especially for Lombardy and the area around Venice [7,8,92]. It is not possible to determine with any certainty whether the glasses are the result of trade with finished products (e.g., the Cape Stoba shipwreck [99]), raw materials, or cullet (e.g., the Serçe Limani shipwreck [100]). However, in line with the rising mercantile role of Venice between major forces such as the Ottonian Empire, the Byzantines, and the Arabs [101], the major pull of the North Adriatic for long-distance traders is hardly surprising. Perhaps Istrian ports and sites in the hinterland benefited from this success indirectly, trading with Venice or other large centers. However, it is also possible that some of the maritime trade supplied the eastern Adriatic coast directly. Moreover, the more widely known Cape Stoba case and the shipwrecks near Savudrija, Umag, and Poreč carrying Byzantine amphorae indicate that some of the merchants sailed along the Istrian coast as well [102]. The dating of the plant ash glass compositions from Koper is not precise but revolves around the 10th or 11th c. In a wide sense, then, the plant ash glasses arrived in the same time window as the much more strongly represented reused old natron glass. The typology of plant ash glasses is more difficult. The pushed-in bases (nos. 1 and 3–5) most probably represent beakers. They are incompletely preserved, so the base diameters cannot be determined with certainty. The bases could also belong to small flasks or bottles, like the rim of sample no. 16. In this case, if they arrived as complete vessels, they may have represented containers, perhaps for precious liquids or perfumes. The shape of the partially preserved neck with a bulge (no. 2) is analogous to the bottles in Syro-Palestine since the Umayyad period and became particularly popular in the 10th and 11th centuries [103]. In Europe, bottles with bulges on the neck appear from the 13th c. onwards [26,104]. The pushed-in bases (in contrast to the merely concave earlier form) appear in Europe around the 12th–13th c. [26], but they are already present in Umayyad contexts in Jerusalem [105] and continued into the following centuries. Based on typology alone, then, an occasional import of vessels, perhaps even as containers, seems more likely than a very early production of these vessel forms.

## 5. Conclusions

Glass from Koper exhibits two major groups. One is natron-type glass according to the Roman tradition, though it has properties specific to Late Antiquity and Early Middle Ages. The most numerous is type *Foy Série 2.1* glass (nine samples: eight goblet feet and one lamp or balsamarium). This type of glass includes a Cunja 2 type and a 9th–11th c. CE thin-stemmed goblet. Two Late Antique goblet feet of small dimensions are made of Levantine sands, attributed to the 6th c. CE Apollonia. Two glasses, a rim, and a lamp handle are of the late 6th–7th c. CE-type Magby, and the composition of the two goblets cannot be determined but points to Egyptian origin. Such a composition shows the predominance of Egyptian glass, which has also recently been confirmed for Italy [106] and the Balkans [64].

It is interesting to note an absence of unaltered HIMT glass, which is normally more frequent in western Europe [107], and a small percentage of the high-quality *Levantine* glass, which is unusual in the Adriatic cities. Glass *Foy Série 2.1* was imported to the Balkans mostly along the major rivers Danube and Sava and from the Aegean ports [40,64]. This is certainly not an optimal way for Koper, and we may rather imagine maritime trade through the Adriatic, as in the case of Croatian islands [108]. This trade might have also continued inland for a certain distance until Korinjski hrib, for instance, which also contains a considerable fraction of *Foy Série 2.1* glass [109].

The second group of six samples is composed of halophytic plant ash of the type *alume catino*, harvested in the Levant. The siliceous component shows rather uniform properties, yet its titanium and zirconium concentrations exclude its Venetian origin. The present calculation also excludes imports from Egypt but points towards Lebanon and Syria, with a small probability also to Iraq and Iran. The glass might have been imported from there as vessels or containers; the other possibility is that the glass vessels were made in (Venetian?) workshops from imported raw glass or cullet. In any case, they seem to predate the ubiquitous glass of the Renaissance period and give an important insight into the very rare presence of Islamic glass on the Adriatic coast.

The role of Egyptian glassworks in the period of Islamic glassmaking is unclear. A shipwreck on the Israel coast loaded with glass cullet testifies to contact with Egypt [110]. According to [46], the Egyptian glassworks were overloaded by the production of architectural glass for the monumental mosques since the end of the 7th c. CE.

Three glasses are out of this scheme. A beaker base was made of plant ash alkalis. Still, its aluminum content suggests Mesopotamian origin, yet its specifics include high manganese content (no. 3). Though its precise attribution is not so clear, it matches very well with traded glass, such as that found in the shipwreck of Serçe Limani. One lamp handle was made of mixed alkalis (composed of natron and plant ash), while its siliceous component is likely of Egyptian origin (no. 20), in accordance with the Magby glass. The third sample, a beaker base, was made of natron glass, but it shows a high aluminum content, pointing towards some other, not yet determinable provenience (no. 11).

Our analysis confirmed the reuse of old natron glass for Early Medieval vessels and for the 9th–11th c. CE thin-stemmed goblet, as has previously been observed in northern, central, and southern Italy [7,9,11,12,23,111]. It is characteristic that all natron glass of Egyptian provenience is recycled. A small presence of non-recycled glass (three samples or roughly 20%) is of Levantine or (in one case) Mesopotamian origin and suggests a modest supply of fresh glass from this region during Late Antiquity.

The goblet feet of larger dimensions were made of the following glass types: Foy 2.1 (6), Levantine I (Apollonia; 2), Magby (1), and an undeterminable glass of Egyptian origin (2). All these types represent natron glass, which, in the literature, is dated from the end of the 5th c. CE (*Foy 2.1*) to the 7th century (*Foy 2.1* and Magby) until the 9th century (*Egypt 2 > 815 CE*). In Koper, this type of glass circulated for two centuries longer, as the last object made of (recycled) natron glass can be dated to the 10th–11th century CE. The glass market re-intensifies after the 10th c. CE, with the influx of plant ash glass from the Syrian and neighboring glassworks.

From a methodology point of view, the Euclidean distance method proved effective and discriminative enough for natron-type glass. For plant ash glass, it seems less selective, probably on account of the larger dispersion of the plant ash elemental concentrations, which also partly overshadow the elements of the siliceous component. A solution may be an improved database with recalculated standard deviations based on critically evaluated experimental data.

## Figures and Tables

**Table 1:** Description of the samples with approximate typological (C2—type Cunja 2) and available stratigraphic dating. Small letters distinguish samples with the same temporary inventory number.

No.	ID	Description	Color	Archaeological Dating
1	626	vessel base	aqua	Middle Ages?
2	429 a	bottle neck	aqua	Middle Ages?
3	429 b	vessel base	brown	indeterminable
4	429 c	vessel base	aqua	Middle Ages?
5	429 d	vessel base	aqua	Middle Ages?
6	624	goblet foot	aqua (patina)	Early Middle Ages?
7	646	goblet foot	greenish (patina)	Early Middle Ages?
8	633	goblet foot	aqua	Late Antiquity
9	695	vessel fragment	aqua	Early Middle Ages?
10	601	goblet foot	aqua	Early Middle Ages?
11	447	beaker base	indeterm. (patina)	Antiquity/Late Antiquity
12	619	lamp/balsamarium	aqua	LA/EMA
13	151	goblet foot	aqua	Late Antiquity
14	594	goblet stem	aqua	EMA/MA
15	715	goblet foot	aqua	Early Middle Ages?
16	417 a	rim of a small bottle	indeterm. (patina)	Middle Ages?
17	417 b	vessel fragment	aqua	Early Middle Ages?
18	875	goblet foot	aqua	Late Antiquity?
19	647	goblet foot	greenish (patina)	Early Middle Ages?
20	186	lamp handle	aqua (patina)	Late Antiquity?
21	170 a	goblet rim	aqua	Early Middle Ages
22	170 b	goblet stem and foot	greenish (patina)	Early Middle Ages (C2)

**Table 2:** Oxide concentrations in the analyzed glasses. The first 11 columns report the oxide concentrations in mass %, and the last 8 columns report the oxide concentrations in µg/g. Single zeros denote values below the detection limit.

	Na_2_O	MgO	Al_2_O_3_	SiO_2_	SO_3_	Cl	K_2_O	CaO	TiO_2_	MnO	Fe_2_O_3_	CuO	ZnO	Br	Rb_2_O	SrO	ZrO_2_	BaO	PbO
1	10.7	3.75	0.93	66.2	0.43	0.77	2.44	13.6	0.106	0.27	0.65	57	127	33	14	841	241	0	95
2	12.1	3.03	0.79	67.3	0.43	0.84	2.34	12.2	0.087	0.18	0.63	29	83	61	19	866	271	0	12
3	9.65	2.44	3.21	67.3	0.33	0.83	2.27	11.8	0.070	1.47	0.47	16	34	55	27	937	32	446	0
4	11.7	3.61	0.91	66.1	0.48	0.86	2.33	13.2	0.119	0.08	0.52	39	157	59	12	837	299	0	58
5	11.2	2.99	0.90	67.3	0.63	0.92	1.79	13.4	0.098	0.03	0.64	19	348	49	12	791	209	0	15
6	17.3	1.62	2.37	66.3	0.81	1.00	0.83	6.94	0.183	0.98	1.02	1300	74	7	8	645	117	0	2230
7	16.7	1.78	2.51	66.8	0.63	0.79	0.79	6.63	0.203	1.13	1.17	1950	96	10	9	590	125	0	4830
8	14.8	0.84	3.08	67.6	0.37	0.91	1.52	10.1	0.108	0.02	0.60	13	11	5	11	609	53	0	30
9	18.5	0.98	2.56	65.0	0.71	0.95	0.78	7.32	0.218	1.22	1.19	1290	62	13	5	697	136	0	2470
10	14.0	1.57	2.32	68.4	0.45	0.96	0.90	9.85	0.267	0.20	0.95	430	35	7	5	319	198	0	172
11	11.7	1.09	7.21	68.5	0.26	0.24	0.95	7.24	0.215	0.67	1.42	1050	69	10	7	588	145	0	2770
12	18.6	0.85	2.26	66.4	0.58	1.09	0.87	7.61	0.141	0.69	0.81	355	36	7	11	550	72	0	438
13	17.2	0.96	2.97	69.1	0.56	0.69	0.55	7.40	0.083	0.02	0.39	4	6	3	8	567	58	0	8
14	17.5	0.86	2.43	67.4	0.55	1.03	0.80	7.49	0.132	0.70	0.82	649	55	7	9	624	79	0	703
15	17.6	1.27	2.48	66.2	0.74	1.09	0.71	7.21	0.190	0.99	0.94	1070	54	10	9	580	102	0	2620
16	11.9	3.27	0.78	66.2	0.61	0.99	1.82	13.3	0.125	0.18	0.61	14	594	53	10	930	326	0	63
17	17.6	0.85	2.48	66.2	0.88	0.87	0.84	7.10	0.153	0.82	0.95	3030	177	15	0	649	39	0	6000
18	15.2	1.21	2.32	67.7	0.52	0.97	0.75	9.23	0.259	0.53	1.05	399	78	4	11	399	187	0	1150
19	16.7	1.21	2.16	65.8	1.14	1.07	0.79	7.58	0.149	0.90	1.05	3190	102	11	19	656	49	0	7160
20	18.3	2.10	1.65	64.8	0.64	0.70	1.69	8.85	0.171	0.13	0.83	12	17	4	9	845	138	0	19
21	16.6	1.52	2.41	66.7	0.66	0.92	1.11	8.04	0.173	0.55	0.91	895	96	4	7	522	123	0	1980
22	17.5	1.39	2.51	68.0	0.72	0.98	0.66	5.88	0.157	0.80	0.76	576	36	9	2	566	94	0	2110

**Table 3:** Glass types of natron glass applied in the numerical classification of the measured glasses according to Equation (1), with their reference mean concentrations and standard deviations. Data were taken from the compilations [46] (1, 4, 7, 8, 11–13, 16, 17, 19), [5] (2, 3), [67] (9, 10), [58] (5, 6), [69] (14, 15), and [44] (18; averaging 11 individual data).

		Na_2_O	MgO	Al_2_O_3_	SiO_2_	K_2_O	CaO	TiO_2_	MnO	Fe_2_O_3_
1	Roman Sb	18.7 ± 1.3	0.41 ± 0.11	1.91 ± 0.21	71.4 ± 1.8	0.45 ± 0.09	5.53 ± 0.84	0.06 ± 0.02	0.01 ± 0.01	0.36 ± 0.1
2	Roman Mn (Britain)	18.31 ± 2.09	0.67 ± 0.14	2.32 ± 0.17	69.62 ± 2.62	0.74 ± 0.14	6.66 ± 1.06	0.10 ± 0.03	0.99 ± 0.12	0.59 ± 0.17
3	Roman Mn (Italy)	15.18 ± 0.84	0.57 ± 0.10	2.59 ± 0.13	70.29 ± 1.08	0.51 ± 0.07	7.83 ± 0.3	0.07 ± 0.01	1.39 ± 0.21	0.20 ± 0.16
4	Roman Mn	16.1 ± 1.3	0.54 ± 0.10	2.62 ± 0.24	69.6 ± 2.3	0.65 ± 0.23	7.92 ± 0.76	0.07 ± 0.02	0.74 ± 0.56	0.4 ± 0.15
5	HIMTa	18.33 ± 1.21	1.05 ± 0.18	2.99 ± 0.33	65.43 ± 1.44	0.47 ± 0.14	6.30 ± 1.02	0.43 ± 0.15	1.92 ± 0.57	1.79 ± 0.38
6	HIMTb	18.25 ± 0.11	1.17 ± 0.12	3.31 ± 0.25	63.8 1± 0.55	0.40 ± 0.03	5.70 ± 0.24	0.54 ± 0.07	1.69 ± 0.16	3.81 ± 0.22
7	Foy Série 3.2	19.0 ± 1.1	0.64 ± 0.21	1.94 ± 0.19	68.1 ± 1.7	0.47 ± 0.16	6.61 ± 0.86	0.10 ± 0.03	0.83 ± 0.27	0.68 ± 0.16
8	Foy Série 2.1	17.7 ± 1.3	1.12 ± 0.25	2.53± 0.23	65.7 ± 1.7	0.75 ± 0.19	8.12 ± 0.92	0.15 ± 0.02	1.41 ± 0.44	1.16 ± 0.5
9	Jalame Mn	15.89 ± 0.85	0.59 ± 0.10	2.69 ± 0.15	68.4 ±1.36	0.80 ± 0.08	8.77 ± 0.46	0.08 ± 0.02	1.93 ± 1.11	0.47 ± 0.08
10	Jalame no Mn	15.74 ± 0.81	0.60 ± 0.15	2.70 ± 0.13	70.55 ± 1.18	0.76 ± 0.12	8.77 ± 0.71	0.08 ± 0.02	0.11 ± 0.09	0.38 ± 0.06
11	Jalame	15.7 ± 0.9	0.59 ± 0.12	2.73 ± 0.17	69.9 ± 1.6	0.78 ± 0.13	8.74 ± 0.67	0.09 ± 0.02	0.65 ± 0.94	0.44 ± 0.19
12	Apollonia (Lev. I)	14.2 ± 1.1	0.68 ± 0.28	3.25 ± 0.18	71.2 ± 1.4	0.62 ± 0.19	8.43 ± 0.79	0.09 ± 0.02	0.02 ± 0.005	0.50 ± 0.11
13	Bet Eli’ezer (Lev. II)	12.3 ± 1.2	0.59 ± 0.12	3.38 ± 0.3	74.4 ± 1.5	0.48 ± 0.08	7.35 ± 0.7	0.11 ± 0.03	0.02 ± 0.004	0.69 ± 0.24
14	Egypt I	18.25 ± 1.38	0.93 ± 0.14	4.05 ± 0.29	70.05 ± 1.21	0.40 ± 0.11	3.03 ± 0.23	0.50 ± 0.12	0.051 ± 0.007	1.74 ± 0.28
15	Egypt II	17.26 ± 1.96	0.58 ± 0.13	2.19 ± 0.35	67.85 ± 1.90	0.32 ± 0.24	9.34 ± 1.27	0.27 ± 0.06	0.03 ± 0.015	0.98 ± 0.23
16	Egypt 2 (<815)	16.5 ± 1.0	0.47 ± 0.09	2.00 ± 0.31	69.7 ± 1.9	0.33 ± 0.09	8.51 ± 1.32	0.20 ± 0.03	0.045 ± 0.083	0.84 ± 0.31
17	Egypt 2 (>815)	13.4 ± 0.6	0.70 ± 0.15	2.52 ± 0.20	70.1 ± 1.4	0.51 ± 0.25	9.57 ± 0.54	0.27 ± 0.03	0.44 ± 0.47	1.18 ± 0.32
18	High Al	16.34 ± 1.74	1.14 ± 0.22	6.08 ± 2.30	62.38 ± 3.67	1.57 ± 0.37	8.38 ± 2.31	0.29 ± 0.25	1.22 ± 0.69	1.02 ± 0.52
19	Magby	16.3 ± 1.3	1.87 ± 0.25	2.03 ± 0.29	65.1 ± 1.7	1.54 ± 0.28	9.09 ± 0.78	0.17 ± 0.03	1.25 ± 0.92	1.27 ± 0.41

**Table 4:** Characterization of the measured samples according to the Al_2_O_3_/SiO_2_ vs. TiO_2_/Al_2_O_3_ diagram (Figure 6) and according to the calculation regarding Equation (1). In columns 3 and 5, the nearest type and the respective Euclidean distance to the group centroids are given. Foy 2.1 is a shorthand notation for *Foy Série* 2.1. The determined glass type implies approximate dating.

No.	Type	Type	Recycling	d	Compositional a Dat
	(Figure 6)	(Calculated)	Markers	(Equation (1))	Dating
1	plant ash	Tyre		0.803	10th–11th c.
2	plant ash	Raqqa 1		0.638	10th–11th c.
3	plant ash	Tyre/Nishapur		1.227/1.317	10th–11th c.
4	plant ash	Tyre		0.785	10th–11th c.
5	plant ash	Raqqa 1		0.912	10th–11th c.
6	Foy 2.1	Foy 2.1	x	0.534	6th–7th c.
7	Foy 2.1	Foy 2.1	x	0.709	6th–7th c.
8	Levantine I	Apollonia (Lev. I)		1.009	6th c.
9	Foy 2.1	Foy 2.1	x	0.610	6th–7th c.
10	Egypt (?)	Egypt (?)/Maby	x	0.888	?
11	?	Indet./High Al		0.636	?
12	Foy 2.1	Foy 2.1	x	0.449	6th–7th c.
13	Levantine I	Apollonia (Lev. I)		0.668	6th c.
14	Foy 2.1	Foy 2.1	x	0.432	6th–7th c.
15	Foy 2.1	Foy 2.1	x	0.429	6th–7th c.
16	plant ash	Tyre		0.990	10th–11th c.
17	Foy 2.1	Foy 2.1	x	0.364	6th–7th c.
18	Egypt (?)	Magby	x	0.852	?
19	Foy 2.1	Foy 2.1	x	0.376	6th–7th c.
20	Magby	Magby		0.471	late 6th–7th c.
21	Foy 2.1	Magby	x	0.531	late 6th–7th c.
22	Foy 2.1	Foy 2.1	x	0.574	6th–7th c.

## Data Availability

The original contributions presented in this study are included in the article. Further inquiries can be directed to the corresponding author.

## Abbreviations

The following abbreviations are used in this manuscript:

PIXE	Particle- induced X-ray emission
PIGE	Proton-induced gamma-ray emission
PCA	Principle Component Analysis
